# The Fascial Capacitor Model: A Biophysical Hypothesis for the Origin of the Local Twitch Response Within Stacking Fascia

**DOI:** 10.3390/ijms27135901

**Published:** 2026-06-30

**Authors:** Hiroaki Kimura, Tadashi Kobayashi

**Affiliations:** 1 Kimura Pain Clinic, Maebashi 371-0013, Japan; 2 Development of Community Healthcare, Hirosaki University Graduate School of Medicine, Hirosaki 036-8562, Japan; tkoba@hirosaki-u.ac.jp

**Keywords:** fascia capacitor, stacking fascia, local twitch response, fascial hydrorelease, mechanotransduction, hyaluronic acid, piezoelectricity, YAP/TAZ

## Abstract

The local twitch response (LTR) elicited during ultrasound-guided fascial hydrorelease (FHR) is conventionally attributed to dysfunctional motor endplates. Recent observational data from a companion study suggest that LTR events may occur preferentially within stacking fascia—a histologically defined multilayered, densified region of deep fascia—at sites not directly attributable to motor endplate excitation. We propose the Fascial Capacitor Model: stacking fascia can be conceptually modeled as a multilayer biological capacitor in which collagen sublayers may act as electrodes and the interposed densified hyaluronic-acid (HA)-rich loose layer may act as the dielectric, with the LTR hypothesized to reflect a transient electrophysiological discharge when a needle bridges its layers. This biophysical model is grounded in the established molecular and histological architecture of human deep fascia, and the analogy is intended as one of structural isomorphism, rather than complete functional equivalence with engineered capacitor devices. Each premise is independently supported by the primary literature from at least eight research lines spanning roughly seventy years. The apparent gap between estimated bulk discharge voltages and motor neuron threshold is addressed by reconsidering needle-tip geometry and stimulation modality, anchored by the ±6 V triboelectric measurements. The pathological extension of the RC time constant in densified fascia—lengthening by several orders of magnitude and estimated to reach the millisecond range—is supported by empirical evidence from fibrotic extracellular matrices in other connective tissues, while tissue-specific in vivo measurements in fascia remain a future task. The model is positioned as the immediate-phase complement to the Fascial Memory Reset Hypothesis, provides a candidate mechanistic interpretation for intra-procedural symptom relief—an as-yet unquantified clinical observation awaiting formal patient-reported outcome (PRO) measurement in a prospective trial—and yields falsifiable predictions. A direct empirical validation program using insulating-needle recording of spontaneous electrical activity (SEA) is in preparation at the corresponding author’s institution.

## 1. Introduction

The local twitch response (LTR)—a brief, involuntary contraction of a muscle band elicited by needle-based interventions including local injections, dry needling, acupuncture, and ultrasound-guided fascial hydrorelease—has, for more than five decades, served as the operational signature of trigger-point and related neuromuscular interventions [1,2]. Travell and Simons positioned the LTR as a defining clinical feature of myofascial pain syndrome [1]; Hong’s controlled comparison of lidocaine injection versus dry needling established the LTR as a predictor of therapeutic effect [2]; and the modern reformulation of trigger-point physiology within the framework of pain neuroscience continues to treat the LTR as a meaningful endpoint [3]. The dominant mechanistic explanation has long been the integrated trigger-point hypothesis, in which the LTR arises from dysfunctional motor endplates with persistent acetylcholine release, recorded as spontaneous electrical activity (SEA) in the immediate vicinity of the endplate zone [4].

However, an increasing body of imaging-guided observational evidence is becoming difficult to reconcile with a strictly endplate-centred account. In our companion observational study (accepted at Medical Sciences (MDPI) in June 2026) [5], we retrospectively analysed a large video archive of ultrasound-guided FHR procedures and identified 89 LTR events, all of which occurred within stacking fascia—the ultrasound-visible phenotype of densified multilayered deep fascia—at diverse anatomical sites, including both intramuscular regions (where stacking fascia may coexist with or encompass motor endplate-bearing muscle) and extramuscular regions (intermuscular fascia, perineural fascia of the sciatic nerve, and ligamentous or aponeurotic layers). This anatomical distribution invites a re-interpretation, rather than a rejection, of the classical endplate-zone observations [1,2,4]: the original localisation of those observations relied on palpation and needle-EMG signal characteristics rather than on direct in vivo visualisation of either the endplate or the surrounding connective tissue. In the pre-high-resolution-ultrasound era, many such observations may have occurred within stacking fascia that was not yet visualisable; in our cohort, the same palpable, twitch-eliciting phenomenon is now anchored to a structure that can be independently identified by high-resolution imaging. The present model is therefore offered not as a replacement of the foundational seventy-year trigger-point literature but as a structural synthesis that places those observations within the visualisable architecture of densified multilayered deep fascia. In that cohort, the concordance was independent of whether the needle tip was at an extramuscular or intramuscular site, a finding more consistent with stacking fascia as the structural feature associated with twitch occurrence. This finding within the retrospective cohort raises a structural question that the endplate hypothesis is not designed to answer: what is it about densified, multilayered deep fascia in particular that converts a brief mechanical perturbation into a synchronous, single-shot muscle contraction?

The present paper proposes a biophysical answer. We argue that the histologically established architecture of stacking fascia—alternating sublayers of densely packed type I collagen separated by glycosaminoglycan (GAG)-rich loose layers whose HA component aggregates pathologically under chronic load—is structurally isomorphic, by analogy, to a multilayer ceramic capacitor (MLCC), and that the LTR is hypothesized to reflect the macroscopic discharge of this biological capacitor when a needle bridges its layers. The model is presented as a Hypothesis (in the *IJMS* Hypothesis and Theory sense): an explicitly testable, falsifiable framework, calibrated to the molecular-biology scope of this Special Issue, offered as a complement to—not a replacement for—the established Travell–Simons–Hong endplate framework. Our group previously proposed the Fascial Memory Reset hypothesis in this journal (*Int. J. Mol. Sci.* **2026**, *27*, 3720) [6], framing the mid- to long-term effects of FHR on a mechano-epigenetic time scale; the present capacitor model addresses the immediate-phase electrophysical event on a complementary seconds-to-minutes time scale.

The hypothesis that stacking fascia spontaneously discharges electrical activity, and that this discharge underlies a range of myofascial phenomena including referred pain, motor dysfunction, and sympathetic-mediated vasoconstriction, was first proposed by the corresponding author at the 20th MPS Research Meeting (Fukuoka, Japan, 15 October 2017) [7]. The present paper develops the biophysical mechanism—the Fascial Capacitor Model—that underpins this 2017 hypothesis.

The remainder of the paper is organized as follows. Section 2 introduces the Fascial Capacitor Model itself, including a five-stage mechanistic cascade and order-of-magnitude quantitative estimates. Section 3 surveys the convergent evidence from eight independent research lines that support each premise of the model. Section 4 directly addresses the voltage gap objection. Section 5 places the present hypothesis in relation to the Fascial Memory Reset Hypothesis across two timescales. Section 6 considers the clinical correlate of intra-procedural symptom relief, including (Section 6.1) an operational definition of stacking fascia, (Section 6.2) broader mechanistic implications, and (Section 6.3) a systematic comparison with competing hypotheses. Section 7 outlines the direct empirical validation program now in preparation. Section 8 concludes.

## 2. The Fascial Capacitor Model

**Figure 1:**
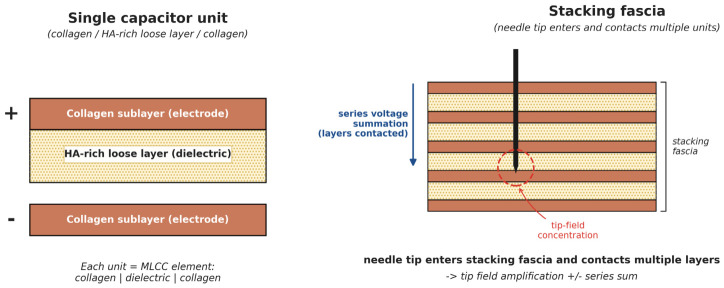
Schematic of the Fascial Capacitor Model. (**Left**) Single capacitor unit: collagen layers (electrodes) sandwich a hyaluronic-acid-rich layer (dielectric). (**Right**) Stacking fascia comprises multiple stacked units; a needle entering the upper portion of the stack contacts and discharges multiple layers, with electric-field concentration at the needle tip.

*Structural analogy.* We emphasize at the outset that the multilayer ceramic capacitor (MLCC) analogy invoked throughout this paper is one of structural isomorphism only—the equivalence concerns geometric layering and dielectric heterogeneity—and does not imply functional equivalence with engineered capacitor devices at the level of charge density, dielectric response, or temporal performance. Direct in vivo measurements of these functional parameters in fascial tissue remain a future task. A multilayer ceramic capacitor (MLCC) stores charge in an alternating stack of conductive electrodes and dielectric insulators. Stecco and colleagues, in their morphometric analysis of human deep fascia, showed that the aponeurotic fascia is built of two to three sublayers of densely packed type I collagen (mean thickness ≈ 277 µm) separated by thinner layers of loose connective tissue (mean thickness ≈ 44 µm), with adjacent collagen layers crossing at approximately 78° [8]. We propose that this histologically confirmed architecture is structurally isomorphic to an MLCC: each collagen sublayer behaves as a relatively conductive electrode (a known piezoelectric medium [9,10,11,12]), and each interposed glycosaminoglycan-rich loose layer, when its hyaluronic acid (HA) component aggregates pathologically (densification [13,14,15,16]), behaves as a dielectric that impedes charge dissipation. Within this framework, “stacking fascia”—the ultrasound-visible (sonographic) phenotype of multilayered, densified deep fascia—corresponds to the physical body of the capacitor (Figure 1).

A five-stage cascade. We propose the following sequence linking chronic mechanical load to symptom generation:

*Stacking formation.* Chronic mechanical stress (repetitive movement, postural overload, post-traumatic immobilization) is associated with an increase in collagen density and HA hyper-aggregation, with cross-linking and reduced water content. The fasciacyte, a fibroblast subtype specialized for inter-layer HA production [17], is a candidate cellular driver of this dielectric remodeling. At the molecular level, fasciacytes are HA-rich extracellular matrix-producing cells, with HAS2 (hyaluronan synthase 2) expression directly quantified in these cells [17]. Under chronic mechanical loading, fasciacytes may shift towards a hyper-secretory phenotype, promoting HA aggregation and extending chain length [13,17,18]. The resulting dehydration and tighter packing between collagen sublayers are hypothesized to progressively transform the loose layer from a hydrated, ion-conducting medium into a low-conductivity dielectric. YAP/TAZ-driven transcriptional programs [19,20,21] further consolidate this fibroblast-to-myofibroblast phenotype, providing the durable mechano-epigenetic substrate of the Fascial Memory Reset Hypothesis [6].

*Capacitor formation.* The alternating arrangement of densified HA between collagen sublayers completes a multilayer capacitor-like structure with measurable charge-storage capacity.

*Charge accumulation and steady-state field.* Daily mechanical loading is hypothesized to generate charge through (a) collagen piezoelectricity [9,10,11,12] and (b) streaming potentials in the GAG matrix [22,23]. In healthy hydrated connective tissue, the dissipative RC time constant (resistance–capacitance time constant) is, on the basis of streaming potential and dielectric measurements of comparable hydrated extracellular matrices [22,23,24], on the order of nanoseconds, so charges do not accumulate over time—consistent with the absence of capacitor-like behavior in healthy fascia. Two complementary mechanisms allow a quasi-steady electrical state to nevertheless emerge specifically in pathologically densified fascia. First, dynamic equilibrium: the body is never mechanically silent, and the continuous low-amplitude loading produced by respiration, cardiac pulsation, postural maintenance, and the friction that accompanies impaired inter-layer gliding sustains a continuous “generation rate”. When this generation rate exceeds the dissipation rate, it supports a non-zero steady-state field. Second, pathological extension of the RC time constant: HA hyper-aggregation and densification of the loose layer dramatically increase its local insulating capacity, with a lengthening by several orders of magnitude across the multilayer structure that is estimated to reach the millisecond range.

This RC extension is empirically supported by impedance studies of fibrotic and densified extracellular matrices in other connective tissues. In hepatic fibrosis, computed time constants progressively elevate with fibrosis grade and electrical impedance characteristics shift correspondingly [25]. Broad-spectrum tissue dielectric measurements demonstrate multi-order-of-magnitude variation between healthy and pathologically remodeled extracellular matrices [24]. These findings do not constitute a direct measurement in fascia; however, the lengthening by several orders of magnitude observed empirically in fibrotic connective tissues in other organ systems supports the parallel proposal for densified fascia, estimated to reach the millisecond range. Tissue-specific in vivo measurement in fascia remains a task for future study. In this regime, physiological mechanical cycles (~0.2–1 Hz) become comparable to or shorter than the dissipation time constant, making quasi-steady-state electric fields physically plausible. The dynamic-equilibrium and RC-extension mechanisms operate concurrently in densified fascia and together account for why capacitor-like quasi-steady-state behavior is hypothesized to emerge preferentially within stacking fascia rather than healthy tissue. Their consequences are detectable as the loss of inter-layer gliding and the increased viscous coupling characteristic of densified fascia.

*Multimodal afferent activation.* The dense innervation of deep fascia [26] is activated through two pathways operating in parallel. First, an electrical pathway: the resulting local electric field acts on voltage-sensitive elements of the free nerve endings (voltage-gated channels and direct depolarization at the tissue–axon interface). Second, a mechanical pathway: the deformation, densification, and chemical microenvironment of stacking fascia mechanically gate Piezo1 and Piezo2 channels [27], which are opened by membrane tension rather than by voltage. TRPV1-positive free nerve endings contribute through their polymodal sensitivity to the chemical and thermal microenvironment of densified fascia. Critically, these pathways are mechanistically distinct—Piezo channels respond to deformation, not to electric fields—but they converge on the same afferent population. Although Fukada–Yasuda shear piezoelectricity strictly requires shear strain across collagen fibrils, the angled needle trajectory across the approximately 78° crossing collagen sublayers [8] is expected to produce fibril-scale shear deformation in addition to bulk compression, with the d_14_ value used as an order-of-magnitude shear estimate. The combined output can be recorded as spontaneous electrical activity (SEA) of the type originally described by Simons [4], but re-interpreted here as arising from fascia per se, rather than from the motor endplate.

*Positive feedback.* Afferent activation may drive pain, referred pain, allodynia, muscle guarding, and sympathetically mediated vasoconstriction. The ensuing ischemia and immobility further promote densification and additional layering, increasing the effective capacitance—a positive feedback loop that may underlie chronicity.

*Quantitative estimates.* Using a parallel-plate approximation, C = *n* · ε_0_ · ε_r_ · A/d, with *n* = 2 dielectric layers (a conservative approximation based on the two-to-three-sublayer architecture reported by Stecco et al. [8]; this treats the alternating sublayer arrangement as effectively parallel during the brief discharge event, an order-of-magnitude approximation appropriate to the Hypothesis-paper scope), ε_0_ = 8.854 × 10^−12^ F m^−1^, ε_r_ ≈ 30 for densified HA (a canonical order-of-magnitude estimate from compiled tissue dielectric measurements for dense connective tissue and similar mid-MHz biological media [24]), d ≈ 44 µm [8], and A ≈ 5 cm^2^ yields a capacitance of the order of C ≈ 6 nF. Taking a collagen piezoelectric coefficient d_14_ ≈ 1 pC N^−1^ [9,10], with nanoscale confirmation [11,12], a steady-state mechanical stress σ ≈ 5 kPa across A ≈ 5 cm^2^ gives a bulk steady-state voltage of order V ≈ 0.4 mV, with stored energy E = CV^2^/2. Under the transient pressure pulse delivered by FHR injection (~200–500 kPa), the same calculation gives a transient bulk voltage of the order V ≈ 25 mV. These are order-of-magnitude estimates intended to bound the plausibility of the model, not point predictions, and they refer to bulk-tissue voltages—see Section 4 for the geometric and modal corrections that apply at the needle tip. A complete derivation, full parameter table with citations, and parameter-by-parameter sensitivity analysis are provided in Supplementary Material S1.

## 3. Convergent Molecular and Biophysical Evidence from Independent Research Groups

A useful test of any biophysical hypothesis is whether each of its independent premises is supported by primary research from groups working without reference to the hypothesis itself. The Fascial Capacitor Model rests on at least eight such independent lines of evidence, accumulated over approximately seventy years.

Collagen piezoelectricity (Fukada–Yasuda research line, Japan). The piezoelectric effect of biological collagen was first demonstrated by Fukada and Yasuda in bone [9] and subsequently characterized in tendon and skin [10]. The piezoelectric coefficient d_14_ is now accepted to lie in the range 0.2–2 pC N^−1^.

Collagen nanoscale electromechanics (Minary-Jolandan and Yu, United States). Atomic-force microscopy measurements of individual type I collagen fibrils confirmed shear piezoelectricity at the nanoscale and excluded a purely surface-charge artefact [11,12], directly relevant to the single-fibril scale at which the proposed mechanism operates.

Streaming potentials in GAG matrices (Grodzinsky group, MIT). The same group that defined the streaming-potential framework for articular cartilage [22] later extended its biomechanical formalism to connective tissue more broadly [23], providing an empirically grounded route by which load-driven fluid flow through a charged HA-rich matrix can generate millivolt-range potentials.

Fasciacyte, HA and densification (Stecco group, Padova). The Padova group identified the fasciacyte as the HA-producing fibroblast subtype of fascia [17], characterized the morphometry and innervation of human deep fascia [8,14], and clarified the role of HA hyper-aggregation in myofascial pain [18]. Pavan and colleagues drew the explicit operational distinction between densification (a reversible HA-driven viscosity change of the loose layer) and fibrosis (an irreversible structural change of the collagen layer) [13]. Fede et al. extended this work to a molecular-biology level in *IJMS* itself [15] and across multiple organ systems [16].

YAP/TAZ mechanotransduction (Dupont/Piccolo group, Padova). The seminal demonstration that YAP/TAZ transduce extracellular matrix stiffness into nuclear transcriptional programs [19] supplies the mechano-epigenetic substrate by which fascial densification is plausibly maintained over chronic time scales [20]. In vivo expression of YAP in human deep fascia has now been directly demonstrated in *IJMS* itself [21], making the present journal the most current scientific substrate for the molecular biology of stacking fascia.

**Piezo** channels as molecular mechanosensors (Coste/Patapoutian group, Scripps). The identification of Piezo1 and Piezo2 as bona fide mechanosensitive cation channels [27] supplies a molecularly defined mechanosensor in fascial fibroblasts and free nerve endings. Piezo channels are gated by membrane tension rather than by voltage, and therefore operate as a parallel pathway alongside, rather than downstream of, any direct electrical events generated by capacitor discharge.

The connective-tissue continuum as a signaling medium (Langevin group, Harvard/NCCIH). Langevin and colleagues established a foundational body of evidence on the connective-tissue continuum as a mechanically responsive signaling medium [28,29,30]. They showed that mechanical signaling propagates through the connective-tissue continuum to coordinate cellular responses across distance [28], and that fibroblasts in connective tissue dynamically remodel their cytoskeleton in response to subcutaneous tissue stretch [29] and to acupuncture-needle stimulation [30]. These findings together support treating the fascial network as a tissue whose biophysical state—mechanical and biochemical, and, by extension within the present model, also electrical—is physiologically meaningful, not merely incidental.

Fascial molecular biology in *IJMS* (Fede, Pirri, and colleagues). Most recently, *IJMS* has itself published primary mechanobiological work on fascia [15,21], establishing a precedent and scope for the present model.

The three classes of charge-generating mechanism—piezoelectricity, streaming potentials, and triboelectricity—operate on distinct time and spatial scales and contribute to the proposed capacitor behavior in complementary, not interchangeable, ways. Collagen piezoelectricity provides continuous low-amplitude charge supply under daily mechanical loading at the molecular scale [9,10,11,12]. Streaming potentials provide additional baseline charge under slow loading cycles (seconds to minutes) at the macroscopic loose-layer scale [22,23]. Triboelectric contact electrification (discussed in Section 4) is a transient surface phenomenon at the metal–tissue interface, activated only during needle insertion and contributing the high-voltage transient that is hypothesized to drive the immediate motor manifestation [31]. The multilayer capacitor structure described in Section 2 acts as the storage and amplification substrate that integrates the slow piezoelectric and streaming contributions and is rapidly discharged at the moment of needle insertion.

Each individual building block of the Fascial Capacitor Model—piezoelectric charge generation, streaming-potential charge generation, HA-driven dielectric behavior, mechano-epigenetic maintenance, and molecularly defined mechanosensors—is independently supported by the primary literature from groups working in unrelated paradigms. The model presented here is therefore best understood as a hypothesis informed by converging evidence from multiple independent literatures.

## 4. Addressing the Voltage Gap

A natural objection to the model is that the estimated bulk voltage produced by capacitor discharge (of the order of microvolts to ~25 mV; Section 2) appears smaller than, or only marginally comparable with, the canonical depolarization required to fire an α-motor neuron action potential (~15–20 mV above resting potential). We address this objection directly, because it rests on three assumptions that are each not fully aligned with the current neurophysiological understanding when the actual neurophysiology and biophysics of needling are considered.

**Assumption 1.** 
*That the local twitch is direct depolarization of a motor neuron*.

This view is not fully consistent with current neurophysiological evidence. The local twitch response (LTR) involves a spinal-reflex pathway, supported by Hong [2] (spinal-cord-section model) and recent reviews [3], in which low-threshold Aδ and group III/IV afferent endings embedded in densified fascia [26] are activated locally by the discharge event; the afferent input is then amplified at the spinal cord through spatial and temporal summation and polysynaptic gain within the interneuron pool, and the amplified signal subsequently crosses the α-motor neuron threshold. The relevant initial excitation threshold is therefore not the somatic threshold of an α-motor neuron, but the much lower threshold of afferent free nerve endings, and the subsequent spinal-cord summation may convert the sub-millivolt afferent signal into supra-threshold motor neuron depolarization. In sensitized tissue, these afferent thresholds are further reduced, as exemplified by Piezo2-dependent mechanosensitization of nociceptors in inflammatory and osteoarthritic conditions [32]. In addition, a direct peripheral coupling pathway—in which the discharge field may also act on peripheral motor axons in the immediate vicinity of the needle tip—operates compatibly with the spinal-reflex pathway; the relative contributions of these compatible pathways constitute an empirically open question to be addressed by the planned SEA validation programme (Section 7). The “15–20 mV” figure of an α-motor neuron, in this context, is applied to the wrong cell type at the wrong point in the pathway.

**Assumption 2.** 
*That the relevant voltage is the bulk-tissue voltage*.

This appears incomplete in light of current neurophysiology. At the needle tip, two well-established geometric and circuit effects amplify the bulk value by approximately one to two orders of magnitude:

*Tip-field concentration.* For a needle with a tip radius of curvature of the order of 10–25 µm, classical electrostatics for sharp conductors predicts that the electric field strength scales steeply with 1/r near the tip, becoming dominant at sub-µm distances. For tip geometries in this range, finite-element and analytical analyses of needle-tip and sharp-electrode field profiles report amplification factors of approximately 10–30-fold at the tip surface relative to the bulk applied potential. The classical 1/r behavior is sufficient on its own to render bulk-tissue voltages inadequate as a measure of the field actually experienced by free nerve endings adjacent to the inserted needle.

*Effective voltage gradients across multiple stacked units in series.* When a needle simultaneously contacts N collagen–HA–collagen sandwich units, those units behave approximately as series capacitors during fast discharge, so the open-circuit voltage scales approximately as N · (Q/C). With allowance for incomplete electrical isolation between layers, a realistic series multiplier is approximately 2–4-fold when several units are engaged.

Even at shallow penetration depths, tip-field concentration provides substantial voltage amplification at the needle tip; an additional contribution from layer summation arises when multiple units are bridged by the inserted needle. The depth of needle insertion at the moment of LTR is typically only into the upper portion of the stacking fascia, consistent with our model in which surface tip-field amplification (and triboelectric contact, see below) constitutes the dominant trigger of discharge. Combined, the tip-field and series effects can yield an effective gain of approximately 30–90-fold as an order-of-magnitude plausibility bound; applied to the 25 mV bulk transient of Section 2, the surface field at the needle tip is of the order of ~1 V as an order-of-magnitude bound, exceeding afferent activation thresholds.

**Assumption 3.** 
*That only direct voltage-mediated depolarization is relevant*.

This assumption likewise overlooks important features of current neurophysiology. Needle insertion is intrinsically a multi-modal stimulus.

*Triboelectric contact electrification.* Chen et al. [31] reported open-circuit voltages of the order of ±6 V in an acupuncture-needle/tissue triboelectric system. In the present manuscript, this finding is used only as an order-of-magnitude empirical anchor for the possible triboelectric component at the metal–tissue interface, not as a direct measurement in human deep fascia during FHR. The triboelectric series for stainless steel against biological polymers has been quantified [33]. The clinical FHR *manoeuvre*—insertion plus micro-rotation of a stainless steel needle through a charged collagen/HA matrix—is the canonical geometry for this transient surface phenomenon at the needle–tissue interface; direct empirical verification in human deep fascia remains a task for the planned SEA validation program.

*Mechanosensitive channels respond to deformation, not voltage.* Piezo1 and Piezo2 are gated by membrane tension [27], with documented contributions to nociceptor sensitization [32]. Their activation by the mechanical component of needle insertion proceeds in parallel with any electrical event.

*Tip-localized electroporation* provides an alternative pathway. Reported electroporation thresholds [34] are reachable in a sub-µm halo at the tip when the gain factors above are applied, providing a route to rapid, transient intracellular calcium (Ca^2+^) cascades in neighbouring fascial cells without classical voltage-gated depolarization. This is a well-characterised biophysical phenomenon distinct from speculative quantum-biological mechanisms.

Once the actual neurophysiology of the LTR (a spinal-reflex pathway with compatible direct peripheral coupling, not direct motor neuron depolarization at the needle tip alone), the actual geometry of the needle tip (~30–90-fold gain), and the actual multi-modality of needle stimulation (triboelectric + piezoelectric + mechanical + chemical) are taken into account, what initially appears as a three-order-of-magnitude “voltage gap” is more accurately described as arising from a mismatch between the level at which the objection is formulated and the level at which the model makes predictions. The model is not required to reach 15–20 mV at the bulk tissue scale, and the relevant stimulus is not a voltage in isolation.

## 5. Integration Across Two Timescales with the Fascial Memory Reset Hypothesis

The model presented here is the immediate-phase complement to the Fascial Memory Reset Hypothesis that we previously proposed [6]. The two operate on fundamentally different time scales and address fundamentally different observables, but converge on the same anatomical substrate.

Within this framework, the local twitch may represent the immediate electrophysical signature of FHR action—interpreted here as the transient discharge of a multilayer fascial capacitor—while the Fascial Memory Reset Hypothesis describes the subsequent mechano-epigenetic remodeling that determines durability of a clinical effect. The two hypotheses are explicitly complementary, not redundant: each addresses observations the other cannot, and together they describe both the millisecond-scale spike (capacitor discharge → LTR) and the long-term substrate reset (mechano-epigenetic remodeling) on a single anatomical structure (stacking fascia).

## 6. Clinical Correlate: Intra-Procedural Symptom Relief

Our companion observational study [5] reports the objective electromechanical sign (local twitch). In clinical practice, a substantial subset of patients additionally report a striking subjective observation: immediate symptom relief—reduction of pain, restoration of range of motion, release of a sensation of “pulling” or “blockage”—during the FHR injection itself, within seconds of needle entry into stacking fascia and bolus delivery. We note at the outset that this intra-procedural relief is presented here as an unquantified clinical observation at this developmental stage of the model, offered as a candidate mechanistic interpretation pending formal patient-reported outcome (PRO) measurement in a prospective trial.

Two conventional explanations have difficulty accounting for the time scale of this observation. Pharmacological explanations are difficult to reconcile with this timescale, given that the injected agent is a buffered Ringer-type solution without anesthetic, anti-inflammatory or neuromodulatory action, and that the relief occurs on a time scale (seconds) inconsistent with diffusion-limited pharmacology. Pure mechanical fluid-dissection explanations are insufficient because they predict a graded effect that should develop over the time required for the bolus to spread and stabilize, not an effectively instantaneous transition.

The capacitor discharge model can plausibly account for this temporal profile through a hypothesized two-phase sequence. Phase 1—the spike: needle entry is proposed to short-circuit the multilayer capacitor, releasing accumulated charge as a transient high-amplitude current pulse that may drive afferent free nerve endings and contribute to the local twitch via the spinal reflex pathway described above. Phase 2—the reset: once the capacitor has hypothetically discharged, any pre-existing steady-state field that may have been driving sensitized afferent input would no longer be present; the field would be, in effect, hypothetically diminished, potentially removing the persistent driver of pain. We therefore tentatively propose that the local twitch (objective sign) and intra-procedural symptom relief (subjective sign) may jointly arise as two complementary observations of the same hypothesized discharge event. We emphasize that this two-phase formulation is a candidate mechanistic interpretation rather than an established sequence, and direct empirical validation through the SEA recording programme described in Section 7 is required. Within this candidate framework, the “Reset” terminology of the related long-term-phase hypothesis [6] may be provisionally extended into the immediate-phase time scale, pending empirical confirmation.

### 6.1. Operational Definition and Independent Identification of Stacking Fascia

The term “stacking fascia” was originally proposed by one of us (T.K., 2021) within the Japanese-language hydrorelease textbook tradition [35] and was subsequently codified in the international fascia literature, culminating in BOX 7.23.1 of our chapter [36] (Kobayashi T., Kimura H., Zenita Y., Imagita H.) in the international fascia textbook edited by Schleip, Stecco, Driscoll, and Huijing. The operational sonographic criteria adopted in the present hypothesis paper for the identification of stacking fascia are anchored in BOX 7.23.1 Essential Criterion 2 of the JNOS Fascial Pain Syndrome (FPS) classification [35,36] and the canonical Padova morphometric reference [8].

BOX 7.23.1 Essential Criterion 2 [36] verbatim states: “Identification of high echoic strip-shaped lesions (stacking fascia) as high-density, adhesive, or cohesive fascia on ultrasound findings on/in the same tenderness spot.” This criterion is operationalized in our clinical practice through four independent sonographic features: (i) two or more sonographically distinguishable hyperechoic strip-shaped sublayers—typically three to five in chronic conditions [35]—separated by hypoechoic loose interlayers; (ii) sublayer thicknesses approximating the Stecco morphometric range (collagen ≈ 277 µm; loose layer ≈ 44 µm per stacking unit) [8]; (iii) loss of inter-layer gliding on dynamic ultrasound, reflecting the loss of fascial sliding described in [35]; and (iv) increased thickness of the low-echogenicity loose connective tissue layer, reflecting cohesion-induced densification with hyaluronic acid hyper-aggregation [7,35]. Critically, BOX 7.23.1 structurally separates stacking fascia identification (Essential Criterion 2—a required item) from local twitch response observation (Confirmatory Observation 2—a supportive item) within the same framework. The present manuscript reflects this structural separation directly: stacking fascia is established prior to and without reference to LTR observation, anchoring the structural premise of our model in this independent classification framework. The robust raw inter-rater agreement reported in the companion study [5] (initial inter-rater agreement 86/90 = 95.6%) is anchored in this independent structural identification.

### 6.2. Broader Mechanistic Implications

While the present paper focuses on local twitch response as the most readily observable manifestation of capacitor discharge, the 2017 framework [7] proposed three distinct downstream consequences of fascial spontaneous discharge:

*Sensory pathway.* Discharge-induced ectopic input into adjacent sensory afferents may contribute to referred pain patterns characteristic of myofascial pain syndrome.

*Motor pathway.* Ectopic input into adjacent motor pathways may contribute to disordered movement and muscle weakness frequently reported in chronic myofascial conditions.

*Sympathetic pathway.* Ectopic input into adjacent sympathetic fibres may underlie vasoconstriction and the chronic pain associated with sympathetically maintained pain syndromes.

These three pathways jointly may contribute to a substantial portion of the clinical heterogeneity of myofascial pain syndrome under a single mechanistic framework. Each represents a falsifiable prediction warranting dedicated investigation in subsequent papers.

### 6.3. Comparison with Competing Hypotheses

While the proposed Fascial Capacitor Model provides a biophysical rationale for the LTR within stacking fascia, several competing or complementary hypotheses must be systematically considered (Table 1). The conventional integrated trigger-point hypothesis [4] attributes the LTR strictly to motor endplate dysfunction. Alternatively, purely mechanical hypotheses suggest that the LTR arises from mechanical afferent stimulation (e.g., Piezo-channel opening independent of electricity) or focal microtrauma reflex.

A critical differentiating factor among these hypotheses is their ability to account for the intra-procedural symptom relief (the immediate cessation of pain/tension within milliseconds to seconds). Pharmacological or neuromodulatory (acupuncture-like) mechanisms typically require minutes to hours to manifest systemic effects, and purely mechanical trauma would likely increase nociception rather than suppress it. Among the competing hypotheses considered here, the Fascial Capacitor Model provides the most direct candidate mechanism for this immediate temporal profile by proposing the macroscopic “collapse” or “reset” of a pre-existing, sensitizing steady-state electric field, though independent validation against alternative formulations remains required. Most importantly, as outlined in Table 1, these hypotheses yield mutually exclusive, falsifiable predictions regarding the anatomical location and electrophysiological signature of spontaneous electrical activity (SEA), which can be arbitrated by upcoming in vivo recording studies.

## 7. Future Directions: Direct Validation by Insulating Needle SEA Recording

The Fascial Capacitor Model makes a defined set of testable predictions. The most direct test is an electrophysiological recording study now underway at the corresponding author’s institution (Kimura Pain Clinic). The broader research protocol has received institutional review approval (Ethics Committee of Isesaki Municipal Hospital, approval no. 2025-8, approved 25 July 2025).

The validation programme is structured around a controlled comparison between motor endplate vicinity and stacking fascia, recorded in two phases: Phase 1 (needle insertion alone) and Phase 2 (during and after fascial hydrorelease injection), with the same anatomical comparison performed in each phase. Insulated disposable needle electrodes (e.g., NM-125I) are advanced under continuous ultrasound guidance into stacking fascia at sites away from any motor endplate, and into matched endplate-vicinity control sites. Stacking fascia is identified independently of LTR observation using the criteria defined in Section 6.1. Spontaneous electrical activity (SEA) is recorded before and after FHR using a clinical EMG system (e.g., Neuropack MEM-8301), with pre-specified amplitude and duration thresholds, to be finalised after a pilot phase (*n* = 5–10), and with predefined controlled design and artifact discrimination procedures. Ultrasound visualization is used throughout to ensure avoidance of inadvertent intraneural placement, which is independently associated with structural nerve injury [37]. To preserve the Hypothesis-paper scope, no preliminary recording data are reported here; the present paper introduces the validation framework, while results will be reported separately. The design directly parallels—but is anatomically distinct from—the classical intramuscular SEA recordings of Simons and Hong [2,4], allowing the predictions of the fascial capacitor framework (SEA from fascia per se; SEA suppression coincident with the local twitch and field collapse) to be evaluated against the predictions of the endplate framework (SEA only near motor endplates). Beyond the local twitch response, the broader 2017 framework [7] generates testable predictions about referred pain, motor dysfunction, and sympathetic vasoconstriction. Validation studies for each of these pathways are planned as separate investigations.

### Limitations

We acknowledge several limitations of the present work. First, voltage quantification. The numerical estimates given in Section 2 (C ≈ 6 nF; bulk steady-state V ≈ 0.4 mV; bulk transient V ≈ 25 mV under FHR pulse) are order-of-magnitude approximations extrapolated from collagen piezoelectric coefficients and stacking-fascia morphometry; precise values can only be established by direct in vivo electrophysiological measurement, which is the central objective of the planned insulating-needle SEA validation study (Section 7). A full derivation, parameter table, and parameter-by-parameter sensitivity analysis are provided in Supplementary Material S1. Second, the boundary of the capacitor analogy. The MLCC analogy is one of structural isomorphism—collagen sublayers as electrodes, densified HA-rich loose layers as dielectrics—rather than complete functional identity; the actual electrophysical generation in fascia is a composite of triboelectric, streaming-potential, and piezoelectric mechanisms whose relative contributions cannot yet be quantitatively decomposed without independent experimental dissection. Third, uncontrolled comparison. The present hypothesis is motivated by the empirical observation reported in the companion study [5] that the LTRs in that cohort all occurred within stacking fascia; however, a formal controlled comparison with FHR delivered to anatomically non-stacking-fascia sites is outside the scope of either paper and will be required as a future prospective study. We note that the companion study [5] itself acknowledges this limitation in its Section 3, and that the SEA validation programme described in Section 7 is specifically designed to address it through a within-subject paired comparison. Fourth, alternative hypotheses not yet excluded. Plausible alternative mechanisms—including reflex amplification of conventional motor-endplate stimulation in geometrically remote fibres, mechanical microtrauma-induced contraction, and Piezo-channel-driven afferent activation independent of any electrical event—cannot be fully excluded by the present analysis; the planned insulating-needle SEA study is specifically designed to discriminate between these alternatives by recording electrical activity from fascia per se in anatomical sites where motor endplates are absent. Fifth, qualitative clinical correlate. The intra-procedural symptom relief observation discussed in Section 6 remains a qualitative clinical impression and awaits formal patient-reported outcome (PRO) measurement in a prospective trial, for which a planned protocol couples time-stamped LTR observation to standardised PRO measurement at predefined intervals. Sixth, single-institution data. The empirical observations supporting the hypothesis derive from a single specialty pain clinic over approximately ten years; multicenter replication of both the structural observation [5] and the SEA recording prediction (planned) will be needed to establish generalizability. In addition, neural amplification mechanisms in noisy biological systems—such as stochastic resonance, in which background fluctuations can cooperatively cross the firing threshold of nonlinear neural elements [38]—are not quantitatively modeled in the present hypothesis paper and may further contribute to subthreshold signal amplification beyond the spinal-reflex pathway discussed in Section 4.

## 8. Conclusions

We have proposed the Fascial Capacitor Model as a biophysical hypothesis for the origin of the local twitch response (LTR) within stacking fascia. The model proposes that stacking fascia may be conceptualized as a histologically defined multilayer biological capacitor—collagen sublayers as electrodes, densified HA-rich loose layers as the dielectric—and that the LTR may reflect the macroscopic motor manifestation of a hypothesized transient electrophysical discharge across this structure when a needle bridges its layers. The hypothesis is motivated by the empirical observation reported in the companion study [5] that all of the evaluable LTR events in that cohort occurred within stacking fascia, with the concordance independent of whether the needle tip was at an extramuscular or intramuscular site.

The model rests on convergent evidence from at least eight independent research lines (Fukada–Yasuda; Minary-Jolandan and Yu; Grodzinsky; Stecco; Dupont/Piccolo; Coste/Patapoutian; Langevin; Fede/Pirri). Its principal numerical objection—the “voltage gap”—has been addressed through three considerations that are not fully aligned with the assumptions underlying the objection: the spinal-reflex amplification pathway with compatible direct peripheral coupling, needle-tip geometric and series amplification, and the multi-modality of needle stimulation, including direct triboelectric measurement of ±6 V at the acupuncture-needle/tissue interface [31]. The pathological RC time-constant extension—lengthening by several orders of magnitude and estimated to reach the millisecond range—is supported by empirical impedance studies of fibrotic extracellular matrices in other connective tissues, while tissue-specific in vivo measurements in fascia remain a future task. The model is falsifiable: it predicts (i) the existence of SEA recordable from fascia per se, away from motor endplates; (ii) the suppression of such SEA coincident with the local twitch and intra-procedural symptom relief; and (iii) the dependence of both observations on the multilayered, densified architecture of stacking fascia rather than on proximity to an endplate. Each of these predictions diverges from the predictions of the integrated trigger-point hypothesis and is therefore independently testable. Critically, all clinical interpretations advanced here—including the proposed mechanism for intra-procedural symptom relief—remain candidate explanations awaiting prospective validation through a formal patient-reported outcome (PRO) measurement, alongside the planned SEA recording programme.

The Fascial Capacitor Model is positioned as the immediate-phase complement to the Fascial Memory Reset Hypothesis [6], which addresses durability of clinical effect on the mechano-epigenetic timescale. Direct empirical validation by insulating-needle SEA recording is in preparation at the corresponding author’s institution, and a parallel programme of terminological consensus on “stacking fascia” is under discussion by the FRS Task Group. The present paper is offered to the *IJMS* Special Issue “Fascial Anatomy and Histology: Advances in Molecular Biology” as a stand-alone theoretical paper that accompanies the empirical observation paper, in the hope that the two together will help bring the molecular and biophysical biology of fascia into closer dialogue with the clinical phenomenology of fascial therapy.

Together with the empirical observation paper [5] and the Fascial Memory Reset Hypothesis (the paper on long-term mechano-epigenetic remodeling [6]), the present paper is intended to contribute one component of a proposed three-part research programme—observation, immediate-phase mechanism, and long-term substrate—whose convergent empirical test will be sought through the planned insulating-needle SEA validation study. For comprehensive clinical and procedural context of US-FHR—including indications, anatomical target sites, and safety considerations—readers are referred to our previously published textbook [35].

## Figures and Tables

**Table 1:** Systematic comparison of proposed mechanisms for the local twitch response (LTR) and intra-procedural symptom relief.

Hypothesis	Primary Trigger Mechanism	Primary Anatomical Site	Accounts for Immediate Intra-procedural Relief	Falsifiable Prediction (Electrophysiological)
**Integrated Trigger-Point (Endplate)**	Acetylcholine leakage; direct motor endplate irritation	Motor endplate zone (muscle belly)	No (Pharmacological washout requires longer time scales)	SEA is strictly localized to endplates; no LTR originates from pure fascia.
**Mechanical Afferent Stimulation**	Pure mechanical deformation opening Piezo-type ion channels	Ubiquitous (fascia, muscle, or tendon)	Partial (Depends on channel adaptation, unlikely to be instantaneous)	LTR occurs without any accompanying macroscopic electrical discharge.
**Microtrauma Reflex**	Focal structural tissue damage inducing withdrawal reflex	Any innervated tissue	No (tissue trauma typically increases local nociception)	LTR amplitude is proportional to tissue damage; lack of specific target structure.
**Acupuncture Neuromodulation**	Aδ/C-fiber stimulation inducing central descending inhibition	Acupoints/connective tissue network	No (Central neurochemical loops require minutes to hours)	Relief depends heavily on stimulation duration (e.g., needle retention/rotation).
**Fascial Capacitor Model (Current)**	Electrophysical discharge of steady-state field bridging densified layers	Multilayered, densified stacking fascia	Proposed yes, pending validation (Hypothesized instantaneous collapse/reset of the sensitizing electric field)	Extramuscular SEA is recordable directly from stacking fascia; SEA suppression coincides with the LTR.

## Data Availability

No new data were generated for this Hypothesis paper. Data underlying the empirical observation that motivates the hypothesis are reported in our companion observational paper [5].

## Supplementary Materials

The following supporting information can be downloaded at: https://susy.mdpi.com/user/manuscripts/displayFile/835254e0af7038c85632aa687b99713a/supplementary.
